# Presence of the *CYP2B6 516G> T *polymorphism, increased plasma Efavirenz concentrations and early neuropsychiatric side effects in South African HIV-infected patients

**DOI:** 10.1186/1742-6405-7-32

**Published:** 2010-08-19

**Authors:** Verena Gounden, Chantal van Niekerk, Tracy Snyman, Jaya A George

**Affiliations:** 1 Department of Chemical Pathology, Faculty of Health Sciences, University of the Witwatersrand, 7 York Road, Parktown, Johannesburg, 2001, Republic of South Africa; 2 National Health Laboratory Services, Charlotte Maxeke Academic Hospital, Parktown, Johannesburg, South Africa

## Abstract

**Background:**

The 516G > T polymorphism in exon 4 of the *CYP2B6 *gene has been associated with increased plasma Efavirenz (EFV) concentrations. EFV concentrations greater than the recommended therapeutic range have been associated with the increased likelihood of developing adverse CNS effects. The aims of this study were to a) determine the presence of the *516G > T *and other *CYP2B6 *exon 4 polymorphisms in a South African group of HIV-infected individuals b) investigate the relationship between the EFV plasma concentrations, the *CYP2B6 516G > T *polymorphism and the occurrence of CNS related side effects in this group of patients and c) develop and validate a rapid method for determination of EFV in plasma.

**Method:**

Data from 80 patients is presented. Genetic polymorphisms in exon 4 of the *CYP2B6 *gene were identified using PCR amplification of this region followed by sequencing of the amplification products. EFV concentrations were analysed by UPLC-MS/MS. Assessment of the presence of CNS related side effects following EFV initiation were elicited with the use of a questionnaire together with physical examination.

**Results:**

Plasma EFV concentrations displayed high inter-individual variability amongst subjects with concentrations ranging from 94 μg/l to 23227 μg/l at 2 weeks post initiation of treatment. For the 516G > T polymorphism the following frequencies were observed 23% of patients were TT homozygous, 36% GG and 41% GT. The TT homozygous patients had significantly higher EFV concentrations vs. those with the wild (GG) genotype (p < 0.05). Patients who experienced no side effects had significantly lower EFV plasma concentrations vs. the group of patients which experienced the most severe side effects (p < 0.05).

**Conclusion:**

The significant association between the 516G > T polymorphism and plasma EFV concentrations has been demonstrated in this study. A rapid and sensitive method for the measurement of plasma EFV concentration was developed and validated.

## Background

Sub-Saharan Africa bears the greatest burden of HIV infection worldwide with data estimating that one in five adults between the ages of 15-49 years is infected [1]. Currently over 400 000 patients receive anti-retroviral (ARV) therapy at South African state hospitals [1]. Efavirenz (EFV), a non- nucleoside reverse transcriptase inhibitor (NNRTI), forms part of the first line therapy for many of these HIV infected individuals. The ARV experience is relatively new to South Africa in comparison to many developed nations and studies looking at adverse effects of treatment and long-term treatment complications are only now beginning to emerge. Clinical trials have reported central nervous system (CNS) side effects in >50% of patients following commencement of EFV therapy [2]. However no studies in South Africa have investigated EFV plasma concentrations and the incidence of CNS related side effects. The reported side effects range from dizziness and headaches to hallucinations, acute mania and psychosis [2]. In patients commencing therapy for the first time, the development of adverse effects may negatively influence adherence and treatment success. Previous studies have shown that plasma EFV concentrations display a large between subject variability with a coefficient of variance (CV) of up to 118% [3]. Prediction of therapeutic efficacy and the likelihood of developing adverse CNS effects have been associated with plasma EFV concentrations [3,4]. Patients with EFV concentrations of > 4000 μg/l may experience neurological adverse effects more frequently, whilst those with plasma concentrations < 1000 μg/l appear to have a greater risk for emergence of selective drug resistance and treatment failure [3].

The reasons for inter-individual variability in terms of drug related toxicity, drug concentrations and drug efficacy are multifactorial and include differences in gender metabolism, drug compliance, presence of underlying diseases, use of concomitant medications as well as genetic factors [5]. Genetic differences among individuals influence metabolism, distribution and elimination of drugs. EFV is primarily metabolised by the cytochrome P450 isoenzyme *CYP2B6 *in the liver [6]. The *CYP2B6 *gene has been mapped to chromosome 19 [7]. It is 28 kb long and consists of 9 exons [7]. The presence of several polymorphisms present in the gene coding for the enzyme may influence drug metabolism. Previous studies have shown that the allelic variant 516G > T (located in exon 4) is associated with diminished activity of the *CYP2B6 *isoenzyme, increased plasma EFV concentrations together with increased incidence of EFV associated neuropsychological toxicity [4,8]. Rotger *et al *identified significant correlations between the presence of the TT genotype and higher intra and extracellular EFV concentrations and between the presence of the single nucleotide polymorphism (SNP) and increased incidence of fatigue, mood and sleep disorders post initiation of EFV [9]. The allelic variant 516G > T was also shown to have increased prevalence amongst African Americans with studies quoting the frequency of this allele as 30-38% [4,10]. Studies in African populations indicate prevalences varying between 36-60% [10-12].

The aims of this study were three-fold 1) to investigate and describe polymorphisms present in exon 4 of the *CYP2B6 *gene in black HIV infected individuals 2) to investigate the relationship between the EFV plasma concentrations and the presence of *CYP2B6 *exon 4 SNPs with the occurrence of CNS related side effects in this group of patients and 3) develop and validate a rapid method for determination of EFV concentrations in plasma to enable monitoring of drug concentrations in HIV-infected patients.

## Materials and methods

### Sample collection

Participants were recruited from Black South African patients attending the ARV clinic at the Charlotte Maxeke Johannesburg Academic Hospital. Informed consent was obtained from all participants enrolled in the study. Ethical approval for the study was obtained from the Research Ethics Committee, Faculty of Health Sciences, University of the Witwatersrand.

Participants included in the study were all treatment naïve, adult patients who were initiated on the triple therapy regimen of EFV, stavudine and lamivudine. All patients received the same dosage of 600mg EFV nightly. It is the general practice at the ARV clinic to not prescribe EFV for any patients with a current or previous psychiatric condition requiring medication or hospitalisation. At the follow up visit 2 weeks post initiation of therapy blood samples were collected. The time interval of two weeks was chosen as plasma EFV concentrations take 6-10 days to achieve steady state concentrations [2]. It was also to ensure better recall of side effects experienced by patients following initiation of ARVs.

Time of last dose was obtained by patient report. Patients who had not taken their EFV the night before or those who had missed more than two doses were excluded from the study. The use of concomitant drugs and herbal medications (refer to List below for further information and exclusion criteria) which are known to influence plasma EFV concentrations were excluded with the aid of a verbal questionnaire administered to all possible participants, prior to enrolment into the study. Patients, who were pregnant, had evidence of hepatic dysfunction or reported significant alcohol consumption were also not included in the study. Samples from 100 patients were used. Liver function tests, viral load and CD4 analyses are performed routinely on all patients commencing ARV therapy at the clinic.

### List of exclusion criteria

Pregnancy or breast feeding

Previous or current psychiatric disease being treated by a medical practioner

Non compliance (missed more than 2 doses in one month)

Alcohol intake >4 units/day for male and > 3 units/day in females (1 unit = 8 g of alcohol) [13]

Patients taking drugs that potentially may interact with EFV metabolism (i.e Rifampicin, Ritonavir, Carbamazepine, Phenytoin, phenobarbitone, St John's Wort)

Hepatic dysfunction as indicated by:

a) Transaminases > 5-10× the upper limit of normal

b) ALP> 5-10× the upper limit of normal

c) Total bilirubin > 2.5-5× the upper limit of normal [14]

K-EDTA samples were collected from patients 2 weeks after initiation of Efavirenz

The samples were separated by centrifugation at 5000 g for 10 minutes (immediately) after collection. Buffy coats were stored at -20°C until DNA extraction and plasma samples were stored at -70°C until the analysis for EFV levels was performed.

### Analysis of Plasma Efavirenz concentrations

EFV was analyzed by Ultra Performance Liquid Chromatography Quatro micro (UPLC-MS/MS), (Waters, Massachusetts, USA). Samples were extracted using solid phase Weak Cation Exchange cartridges (WCX, Oasis-Microsep, Massachusetts, USA). 200 μl of plasma was used for analysis of the drug concentrations. Chromatographic separation was performed on an Acquity, (Waters, Massachusetts, USA) phenyl column 1.7 μm (2.1 × 50 mm). The chromatographic column used was stable for > 200 injections. The mobile phase consisted of A: B at a ratio of 30:70 (2 mM ammonium acetate with 1% formic acid: 100% Acetonitrile (ACN)) this was run on a gradient with the analyte eluting within 1.5 min. The column temperature was maintained at 50°C throughout the runs. Injection volume for each sample was 10 μl.

The instrument was operated in Electron spray ionization positive (ESI+) mode. The MRM transition used for EFV was *m/z *(mass to charge ratio) [M^+^ACN^+^H]^+ ^357.7 > 316.3. Retention time was 0.72 min with total run time of 2 min.

A standard EP10 evaluation [as per Clinical and Laboratory Standards Institute (CLSI) protocol] to assess recovery, assay precision and linearity was performed for validation. This protocol examines specific performance parameters such as linearity, carryover, bias and recovery [15]. Commercially available calibrator standards and controls were used (Chromosystems Instruments and Chemicals GmbH, Munich, Germany). Calibration curves and controls were run with every batch of patient specimens. The correlation coefficient of the standard curves obtained on multiple days was consistently ≥ 0.98 (n = 18). Separated specimens were stable at 3 months stored at -70°C. No changes were observed in plasma that had been subjected to two freeze-thaw cycles.

### Assessment of EFV-related side effects

Prior to treatment initiation all patients were assessed by a medical doctor to determine the presence of any baseline neuropsychiatric symptoms. Features that were looked for included a previous history of a psychiatric complaint as well as current presence of suicidal ideation, delusions or psychosis. A general neurological exam was also performed on possible participants.

A questionnaire (refer to Additional file 1) adapted from one used in the AIDS Clinical Trials Group study A5095 was administered to all participants at the 2 week follow up post EFV initiation [16]. Responses were scored in terms of frequency of side effects (such as headache, dizziness and other neuropsychological side effects associated with EFV use) experienced and severity in terms of effect on daily activities (severity was scored ranging from no effect on daily activities to unable to carry out daily activities). The maximum score that could be obtained was 72 points. Based on their questionnaires, subjects were grouped into those with no side effects (Group1), those with mild symptoms (1-12 points-Group2), with moderate symptoms (13-48 points -Group3) and with severe side effects (> 48 points or presence of hallucinations or psychotic episodes-Group 4). At the same visit the patients were also examined by a medical doctor for any clinical signs or symptoms of the neuropsychiatric and other EFV related side effects. Patients' clinic files were reviewed post 1 month follow-up to determine the persistence of neuropsychiatric symptoms as per patient complaints and physician assessment

### Further follow up

Viral loads for participants at 3 or 6 months post initiation of therapy were also reviewed using our laboratory information system. A successful viral load response was defined as a viral load below the detection limit of 50 copies/ml.

### Analysis of SNPs

Subjects were genotyped for *CYP2B6 *516G > T (rs3745274). DNA extraction was performed using Invisorb Blood Mini Kit (Invitek, Germany). Forward (5'-TGTTGTAGTGAGAGTTCAATG-3')and reverse (5'-CTATCCCTGTCTCACCGTC-3') primers for exon 4 were designed using the published gene sequence on GenBank (accession number NM 000767) together with the software programme GeneRunner version 3.05 (Hastings Software Inc.). Patient sequences were amplified using conventional PCR. PCR products were run on agarose gels together with 50 bp DNA molecular weight marker (Generuler; Fermentas, Lithuania) and a negative control to detect any possible contamination. Amplicons were sequenced by Inqaba Biotech (South Africa). Sequencing was performed using a Spectrumedix SCE 400 Genetica analysis system (Spectrumedix LCC, USA). Sequences were analysed using the Sequencher program version 4.1.4 (Genecodes, USA).

### Data analysis

The sample size (n = 54) required to detect significant differences in EFV concentrations across the different genotypes with a statistical power of 0.90 was determined. The parameters for an α level (Type 1 error) and effect size were 0.05 and 0.5, respectively. Sample size calculation was performed using the G*Power program, version 3.1.2 (Universität Kiel Dusseldorf, Germany).

The Chi-squared test for the assessment of Hardy-Weinberg equilibrium for the analyzed SNP was performed using software on the Online Encylcopedia for Genetic Epidemiology Studies [17]. All other statistical analyses were conducted using the Statistica program, version 8 (Statsoft, Tulsa, USA). Data was assessed to be parametric using the Shapiro-Wilks W test. One way Kruskal Wallis ANOVA was used to compare EFV concentrations as well as follow-up viral loads across the three genotypes. Spearmen rank order correlation was used to assess the relationship between EFV concentrations and follow-up viral loads.

Multivariate regression analysis was used to demonstrate the relationship between possible confounding variables BMI, age, CD4 count, viral loads and sampling times on plasma EFV concentrations.

## Results

Data for 80 patients were analysed. Twenty patients were excluded due to insufficient plasma volumes for UPLC-MS/MS analysis (n = 1), poor DNA yields following extraction (n = 10) or technical problems with regards to sequencing (n = 9).

The main characteristics of the study cohort are summarised in Table 1.

**Table 1:** Baseline characteristics and summary of findings from data of the 80 patients analysed in the study.

*Variable*	*All*	*GG *	*GT*	*TT*
Age (years)	37.5 (SD:9.0),(n = 80)	38.0 (SD:8.5),(n = 29)	37 (SD:9.0),(n = 33)	33.5 (SD:10.2),(n = 18)

Sex	Male: 20 Female: 60	Male: 8 Female:21	Male:7 Female:26	Male: 5 Female:13

BMI (kg/m^2^)	22.6 (SD: 3.6),(n = 80)	22.4 (SD:3.9), (n = 29)	23.2 (SD:3.4), (n = 33)	22 (SD:3.0),(n = 18)

Initial CD4 count (×10^6 ^l)	128.5 (IQR:142),(n = 80)	113 (IQR:114),(n = 29)	131(IQR:148),(n = 33)	158 (IQR:9),(n = 18)

Initial viral load(copies/ml)	86450 (IQR:2.3 × 10^6^),(n = 76)	96600 (IQR:2.2 × 10^6^),(n = 27)	84900 (IQR:2.4 × 10^6^),(n = 33)	86500 (IQR:2.1 × 10^6^),(n = 16)

Presence of side effects(%)	84 (n = 67) *	76 (n = 22)	85 (n = 28)	94 (n = 17)

EFV concentrations (μg/l)	3980 (IQR:4476),(n = 80)*	2260 (IQR:3411),(n = 29) **	3858(IQR:2385),(n = 33)**	7136(IQR:3623),(n = 18)**

IQR: interquartile range

n = number

BMI = Body mass index

Parametric data displayed as mean (1SD), non-parametric data displayed as median (IQR)

* p value (< 0.05) for Spearmen correlation between EFV plasma concentrations and the presence of side effects (dependent variable)

** p value (< 0.05) for ANOVA analysis of EFV concentrations across genotypes for TT vs. GT/GG

Side effects as per questionnaire score

The genotype distribution and EFV concentrations were as follows: 36% (n = 29) of patients were homozygous GG for the *CYP2B6 516G > T *polymorphism with median EFV plasma concentration of 2260 μg/l (range 94 μg/l to 12957 μg/l); 23% (n = 18) of patients were characterised as homozygous *TT*, had a median EFV concentration of 7136 μg/l (range 1334 μg/l to 23227 μg/l); 41% (n = 33) of patients were heterozygous GT for the polymorphism with a median EFV concentration of 3857 μg/l (range 184 μg/l to 15581 μg/l). The frequency of the 516G > T allele was 43% in our study population. The observed genotype frequency was in Hardy-Weinberg equilibrium

**Figure 1:**
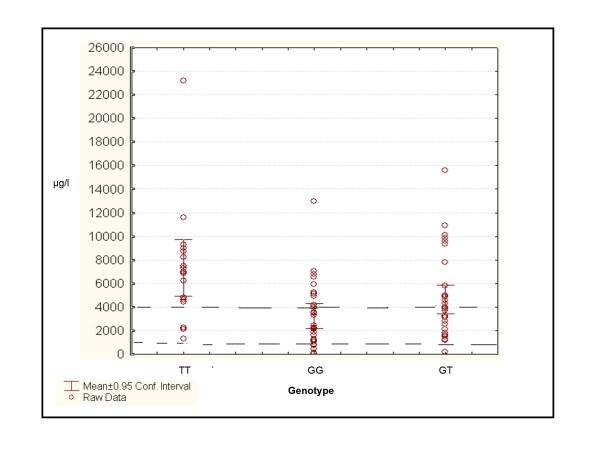
**Dot plot of EFV plasma concentrations by *CYP2B6-516 *genotype**. *GG*, homozygous wild-type; *GT*, heterozygous genotype, *TT *homozygous genotype. --*Value between dashed lines represents therapeutic range*.

**Figure 2:**
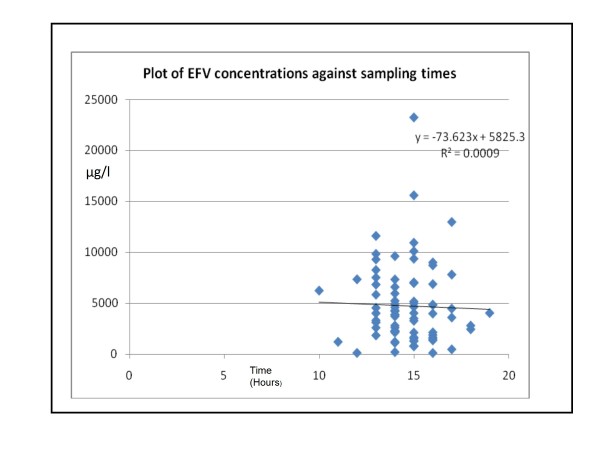
**Plot of EFV plasma concentrations against sampling times**. R^2 ^value of 0.0009 indicates no correlation between sampling times and EFV plasma concentrations of patients involved in the study.

Plasma EFV concentrations in patients ranged from 94 μg/l to 23227 μg/l (median 3980 μg/l), confirming the high inter-individual variability previously noted in patients receiving EFV therapy [3,12]. Only 51% of patients had EFV concentrations within the recommended concentration range of 1000 μg/l to 4000 μg/l [3]. 9% of patients had levels below 1000 μg/l. Interestingly, most (61%) of those who were homozygous GG for the 516G > T polymorphism had EFV concentrations within the therapeutic range, whilst only 16% of those with the TT genotype had concentrations within this range. Plasma EFV concentrations were analysed across genotype groups using a Kruskal-Wallis ANOVA. This demonstrated that patients who were homozygous TT for the 516G > T polymorphism in exon 4 had significantly higher EFV concentrations vs. those patients with the GG or GT genotype (p < 0.05) (refer to Figure 1). The average time between last dose of EFV taken by patients and sample collection was 14.6 ± 1.5 hours. Using simple regression EFV plasma concentrations displayed no significant correlation with sampling times (R^2 ^= 0.0009) (Refer to Figure 2). Multivariate regression analysis also demonstrated that sampling times as well age, BMI, initial CD4 counts and viral loads did not significantly correlate with EFV concentrations of patients (R^2 ^= 0.107, p = 0.23).

**Figure 3:**
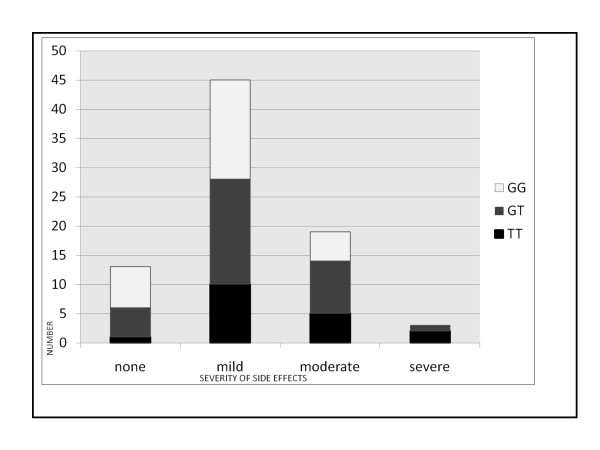
**Distribution of side effects with regards to different *CYP2B6 516G > T *genotypes**. *Based on their questionnaires, subjects were grouped into those with no side effects (Group1), those with mild symptoms (1-12 points- Group2), with moderate symptoms (13-48 points -Group3) and with severe side effects (> 48 points or presence of hallucinations or psychotic episodes-Group 4). Please refer to text for further detail. GG (n = 29): 24% (n = 7) no side effects reported; 59% (n = 17) mild side effects; 17% (n = 5) moderate side effects; none with severe side effects. GT (n = 33): 15%(n = 5) no side effects; 55% (n = 18) mild side effects; 27% (n = 9) moderate side effects; 3% (n = 1) severe side effects TT (n = 18): 5% (n = 1) no side effects; 56% (n = 10) mild side effects; 28% (n = 5) moderate side effects. 11% (n = 2) severe side effects. (Refer appendix for patient questionnaire).

85% of patients experienced some form of EFV-related side effect. The majority of patients, who had experienced side effects following initiation of Efavirenz therapy, had mild symptoms with dizziness (55%) and headache (45%) as the two most frequent complaints. No patients reported suicidal ideation whilst only 5% of patients reported having experienced hallucinations following initiation of EFV therapy. Statistical analysis by Spearmen rank order correlation exhibited a significant correlation (p < 0.05) between questionnaire scores and EFV concentrations amongst participants. The patients who experienced no side effects had a significantly (Analysis by Kruskal Wallis ANOVA p < 0.05) lower median EFV plasma concentration of 2666 μg/l (concentrations ranged from 102.3 μg/l to 4839.7 μg/l) compared to the group which experienced the most severe side effects with a median EFV plasma concentration of 14882 μg/l (concentrations ranged from 9825 μg/l to 23227 μg/l). Refer to Figure 3 for side effect scores as per questionnaire for each genotype. 33% (7 of 21) of all patients who reported severe and moderate EFV related side effects carried the TT genotype. Patients homozygous for the *CYP2B6 516G > T *showed increased overall side effects as compared to those displaying the wild type genotype. However this difference was not statistically significant when Kruskal Wallis ANOVA was performed across the genotypes (p = 0.08). At the 1-month follow-up visit following initiation of therapy, the specific EFV-related side effects had resolved for all patients involved in the study.

We also analysed patient sequences for the presence of other exon 4 SNPs found within the *CYP2B6 *gene namely 503C > T (rs36056539), 593T > C (rs36079186), 499C > G (rs3826711), 546C > G (rs45459594) and 547G > A (rs58871670). None of these polymorphisms were detected amongst our cohort.

Kruskal-Wallis ANOVA showed no significant correlation (p = 0.32) between the GG, TT and GT genotypes and follow up viral loads (performed at 3 or 6 months post initiation of therapy). Spearmen correlation also showed no significant (p = 0.10) relationship between the two week EFV plasma concentrations and the follow up viral loads. 15% (11 of 72 patients for which records of follow up viral loads were available) of patients had viral loads above the detectable limit. These 11 patients' viral loads ranged from 110 to170000 copies/ml. Only 1 of these patients had an EFV concentration lower than the therapeutic level at the initial measurement. Four of these eleven patients had plasma EFV concentrations above the recommended therapeutic range on initial measurement

In terms of method characteristics for the UPLC-MS/MS: the extraction efficiency/recovery ranged from 83-118%, with a mean recovery following extraction 101%. The assay was linear up to a concentration of 30630 μg/l. The limit of detection (LOD) for the assay is 85 μg/l and the limit of quantitation (LOQ) is 101 μg/l. Intra-assay and inter-assay precision CV's ranged from 2.8 to 10%, and 8 -8.9%, respectively. Analysis showed no significant carryover or drift

## Discussion

This study revealed the prevalence of the allelic variant *CYP2B6 *TT (poor metabolisers) to be 23% amongst our study population. The percentage is very similar to the Adult AIDS Clinical Trials Group study by Haas *et al*, which reported a 20% prevalence of the TT genotype amongst their African-American cohort [4]. The authors of the current study also observed the statistically significant (p < 0.05) relationship between the occurrence of severe EFV related side effects and increased plasma concentrations of the drug.

Gatananga *et al *showed that those patients with the *CYP2B6 516G > T *SNP had significantly higher plasma EFV concentrations (> 6000 μg/l) on the standard dosing regimen [18]. In that study the reduction of the initial EFV dosages to either 400 mg or 200 mg resulted in lowering of EFV concentrations towards the therapeutic range and an improvement in CNS related symptoms in the majority of these patients. In our study, the median EFV concentration for the TT homozygotes was 7136 μg/l. It would have been interesting to note, whether in our population, a decrease in dosage would have had a similar effect.

None of the other published SNPs (as mentioned earlier) in exon 4 of the *CYP2B6 *gene were detected in patients from this study. These results are similar to the findings of a study where the frequency of the 503C > T allele was found to be 0% and 2.5% amongst African Americans and Ghanaians, respectively [10]. Both the 503C > T and 593T > C polymorphisms are associated with amino acid changes but their clinical association with EFV concentrations has not been fully elucidated.

In this study a significant relationship was found between the 516G > T SNP, plasma EFV concentrations and increased reporting of CNS side effects. However all patients denied persistence of the CNS symptoms at the 4 week follow-up -post initiation of therapy. It is likely that those with the 516G > T allele still had high plasma EFV concentrations despite improvement of symptoms. Haas *et al *reported increased plasma EFV concentrations in patients with this SNP at 24 weeks post initiation [4]. However in that study, increased CNS symptoms were only reported during the first week following treatment commencement and thereafter patients seemed to develop a tolerance to these side effects despite continued high EFV concentrations. Fumaz *et al*, in a long term follow up of patients receiving EFV therapy demonstrated that more than 50% of the patients had persistent though mostly mild neuropsychiatric symptoms [19]. The presence of other factors associated with the CNS side effects as well as the adequacy of assessment of neuropsychological side effects, needs to be examined [20].

The relationships between drug efficacy and lower virological failure rates when optimal drug concentrations are achieved have been demonstrated in a number of studies [3,8,10]. Repeated exposure to sub-therapeutic concentrations of EFV also increases the chance for the development of resistant viral strains and thus treatment failure [21]. The long half-life of EFV suggests that treatment interruption in patients carrying the TT genotype also selects for EFV resistance due to sub-therapeutic concentrations for extended periods [22]. EFV resistance appears to be relatively common. The K103N mutation associated with EFV resistance was identified in 25% of HIV infected patients with drug resistance in a recent study performed in Johannesburg [23]. In our study 9% of patients had EFV concentrations below the therapeutic minimum of 1000 μg/l which would be a risk for development of EFV resistance in these patients. TDM could be useful in identifying these patients with a view to optimising treatment by either increasing EFV dosages, changing to alternate regimens or identifying non compliance. Poor adherence must also be considered as a cause of sub-therapeutic EFV concentrations in patients. Unfortunately in this study we were only able to assess compliance by patient report, which is often inaccurate and unreliable. Follow up of patient viral load at 3 or 6 months indicated that for the majority of patients initial EFV concentrations had no significant effect on viral suppression. It is possible that patients may achieve adequate viral load suppression on lower doses of EFV than are currently prescribed. However, in this study information regarding change in treatment regimens and patient adherence were not readily available post the one month follow up period of this study. Longer follow-up studies should be done to test this hypothesis.

There are limitations to our study. One limitation is that genotyping for other significant polymorphisms affecting EFV metabolism were not performed. The presence of the *CYP2B6 983 T > C*, although less frequently found in African populations, has also been associated with increased plasma EFV concentrations [24]. Other SNPs in genes coding for metabolizing enzymes such as *CYP2A6*, and *UGT2B7 *have been associated with increased EFV concentrations [25,26]. Pharmacokinetics has shown that trough concentrations of drugs are the most useful in assessing efficacy and toxicity of the drug. The nighttime dosing of EFV results in difficulty obtaining trough doses. The suggested therapeutic range of 1000 - 4000 μg/l is not based on trough concentrations but on concentrations 8-20 hours post dosing [3]. Lopez *et al *demonstrated that trough levels are not estimated with sufficient accuracy when blood samples taken at 8, 12 and 16 hours post dosage were used. This is despite the close linear relationship between plasma EFV concentrations at these time points and trough concentrations [27]. Evidence for the use of this therapeutic range in assessing the relation between treatment efficacy and EFV plasma concentrations has been weak in other studies [28-31]. Twenty percent of participants enrolled in the study were not included in the final analysis. This was largely due to problems with DNA extraction and genotyping. A possible introduction of bias may have occurred by not being able to include data from these patients in the final analyses, although the final sample size obtained was adequately powered.

In nations like South Africa where the goal of adequate access to antiretroviral therapy for all HIV-infected patients is still to be achieved, the added expense of pharmacogenomic genotyping and TDM may seem unrealistic. TDM for EFV using a LCMS/MS methods such as that described in this study allows for accurate measurements and high throughput with a run time of only two minutes. However the evidence that genotyping and measurement of EFV plasma concentrations actually improve patient outcome is lacking. Furthermore in this study, patients' EFV related side effects resolved within a month and there was no significant correlation between patients follow up viral loads and their plasma EFV concentrations. In view of this the authors feel that TDM for EFV therapy may have a role in assessment of patient adherence. However our findings suggest that use of TDM does not improve patient outcomes and larger longitudinal studies are required before a final recommendation can be made with regards to routine implementation of TDM in South African HIV infected patients receiving EFV therapy.

## Abbreviations

HIV: Human immunodeficiency virus; HAART: Highly active ante-retroviral therapy ARV: anti-retroviral; EFV: Efavirenz; NNRTIs: Non-nucleoside reverse transcriptase inhibitors; NRTIS: nucleoside reverse transcriptase inhibitors; HPLC: high performance liquid chromatography; MS: mass spectrometry; PCR: polymerase chain reaction; CNS: central nervous system; TDM: Therapeutic drug monitoring; LOD: Limit of detection; LOQ: Limit of quantitation; μg/l: micrograms/l

## Declaration of competing interests

The authors declare that they have no competing interests.

## Authors' contributions

VG recruited patients for study, administered the questionnaire, examined participants and drafted the manuscript

CN and VG designed primers and optimized PCR for the exon. VG collected samples, extracted DNA and performed PCR on patient samples. CN and VG were involved in analysis of sequencing data.

TS developed the extraction method and UP-LC/MS method for the measurement of EFV in plasma samples. VG and TS were both involved in running patients samples.

JG conceived and designed the study helped to draft the manuscript.

VG performed the statistical analysis.

All authors read, assisted in revision and approved the final manuscript.

## Supplementary Material

Additional file 1 **Side effect questionnaire**. A copy of the questionnaire used to assess the presence of neuropsychiatric side effects post EFV initiation in study participants Click here for file
