# Supplementary material for: Presence of the CYP2B6 516G> T polymorphism, increased plasma Efavirenz concentrations and early neuropsychiatric side effects in South African HIV-infected patients
Source: AIDS Res Ther. 2010 Aug 19;7:32. doi: 10.1186/1742-6405-7-32 (PMC2933581; doi:10.1186/1742-6405-7-32)
Supplement: Additional file 1 — Side effect questionnaire. A copy of the questionnaire used to assess the presence of neuropsychiatric side effects post EFV initiation in study participants [file 1742-6405-7-32-S1.DOC]

**Appendix. PARTICIPANT QUESTIONNAIRE FOR EFAVIRENZ RELATED SIDE EFFECTS**

Thank you for your participation. The purpose of this questionnaire is to help us evaluate the side effects you may have experienced since you started the ARV treatment.

Did you experience any of the side effects listed below, after starting the ARV treatment? Please circle your answer box.

**1. Dizziness…………………………………………… YES (1) NO (0)**

a) How often did you experience this problem in one week? ONCE (0) TWICE (1) THRICE (2) MORE THAN THREE TIMES A WEEK (3)

b) Did it interfere with your daily activities/work? NEVER (0) SOMETIMES (1) MOST TIMES (2)

**2. Difficulty sleeping………………………………….. YES (1) NO (0)**

a) How often did you experience this problem in one week? ONCE (0) TWICE (1) THRICE (2) MORE THAN THREE TIMES A WEEK (3)

b) Did it interfere with your daily activities/work? NEVER (0) SOMETIMES (1) MOST TIMES (2)

**3. Headache…………………………………………… YES (1) NO (0)**

a) How often did you experience this problem in one week? ONCE (0) TWICE (1) THRICE (2) MORE THAN THREE TIMES A WEEK(3)

b) Did it interfere with your daily activities/work? NEVER (0) SOMETIMES (1) MOST TIMES (2)

**4. Difficulty concentrating…………………………….YES (1) NO (0)**

a) How often did you experience this problem in one week? ONCE (0) TWICE (1) THRICE (2) MORE THAN THREE TIMES A WEEK(3)

b) Did it interfere with your daily activities/work? NEVER (0) SOMETIMES (1) MOST TIMES (2)

**5. Scary dreams ……………………………………. YES (1) NO (0)**

a) How often did you experience this problem in one week? ONCE (0) TWICE (1) THRICE (2) MORE THAN THREE TIMES A WEEK(3)

b) Did it interfere with your daily activities/work? NEVER (0) SOMETIMES (1) MOST TIMES(2)

**6) Vivid dreams……………………………………..YES(1) NO(0)**

a) How often did you experience this problem in one week? ONCE (0) TWICE (1) THRICE (2) MORE THAN THREE TIMES A WEEK(3)

b) Did it interfere with your daily activities/work? NEVER (0) SOMETIMES (1) MOST TIMES(2)

**7) Confusion…………………………………………. .. YES(1) NO (0)**

a) How often did you experience this problem in one week? ONCE (0) TWICE (1) THRICE (2) MORE THAN THREE TIMES A WEEK(3)

b) Did it interfere with your daily activities/work? NEVER (0) SOMETIMES (1) MOST TIMES (2)

**8) Did you feel sad/depressed (crying a lot, poor appetite, poor sleep)**

**YES(1) NO(0)**

a) How often did you experience this problem in one week? ONCE (0) TWICE (1) THRICE (2) MORE THAN THREE TIMES A WEEK(3)

b) Did it interfere with your daily activities/work? NEVER (0) SOMETIMES (1) MOST TIMES (2)

**9) Nausea …………………………………………….YES (1) NO(0)**

a) How often did you experience this problem in one week? ONCE (0) TWICE (1) THRICE (2) MORE THAN THREE TIMES A WEEK(3)

b) Did it interfere with your daily activities/work? NEVER (0) SOMETIMES (1) MOST TIMES (2)

**10) Vomitting…………………………………………..YES(1) NO(0)**

a) How often did you experience this problem in one week? ONCE (0) TWICE (1) THRICE (2) MORE THAN THREE TIMES A WEEK(3)

b) Did it interfere with your daily activities/work? NEVER (0) SOMETIMES (1) MOST TIMES (2)

**11) Abdominal pain…………………………………..YES (1) NO(0)**

a) How often did you experience this problem in one week? ONCE (0) TWICE (1) THRICE (2) MORE THAN THREE TIMES A WEEK(3)

b) Did it interfere with your daily activities/work? NEVER (0) SOMETIMES (1) MOST TIMES (2)

**12) Rash…………………………………………………YES (1) NO(0)**

**15) Hallucinations (seeing things other people can't see) YES NO**

**16) Suicidal tendencies requiring admission to hospital... YES NO**

**17) Mental disturbances that required admission to hospital. YES NO**

**18) Are you taking any other medication, herbal or traditional medicine YES NO**

If YES what are the names

**19). Do you drink alcohol? YES NO**

if yes how many drinks do you have a day 1 / 2 / 3 / 4/ more than 4

a“drink” is equivalent to…

• 340 ml beer (1 can of beer)

• 125 ml wine (half a normal glass, one wine glass)

• 25 ml spirits (whisky, brandy, etc.) (1 shot)

• 60 ml sherry

• 25 ml liqueur (1 shot)

Appendix to questionnaire

Points are allocated to each possible answer as indicated above

-No symptoms (0)

-Mild symptoms –with a total score of (1-12)

-Moderate symptoms- with a total score of (13-48)

-Severe symptoms-a score of >48 or the presence of hallucinations,

-suicidal tendencies or hospital admissions for mental disturbances
